# A Malignant Granular Cell Tumor Excised with Mohs Micrographic Surgery

**DOI:** 10.1155/2012/453569

**Published:** 2012-03-01

**Authors:** David Crowe, Elias E. Ayli, Hugh M. Gloster

**Affiliations:** Department of Dermatology, University of Cincinnati College of Medicine, Cincinnati, OH 45267, USA

## Abstract

Malignant granular cell tumors are extremely rare, aggressive neoplasms displaying rapid growth and frequent associated metastatic disease. Excision and evaluation for metastatic disease are mandatory. We present a 54-year-old patient with a malignant granular cell tumor, treated with Mohs micrographic surgery. Cutaneous granular cell tumors are uncommon neoplasms, likely of perineural origin. Most follow a benign and uneventful course, with wide local excision being the treatment of choice (Enzinger, 1988). The malignant granular cell tumor is an extremely rare, aggressive variant, which provides a diagnostic challenge and management dilemma, especially with early presentation when it may be mistaken for other entities. There is also controversy regarding surgical management and follow-up of both benign and malignant granular cell tumors.

## 1. Case Report

A 54-year-old African American female presented with a 1-year history of an asymptomatic, rapidly growing lesion on the right side of her neck. On physical examination, there was a 3 cm, firm, mobile, slightly hyperpigmented, subcutaneous nodule involving the right lateral surface of the neck. The differential diagnosis included epidermoid cyst and adnexal tumor. An incisional biopsy was performed.

**Figure 1:**
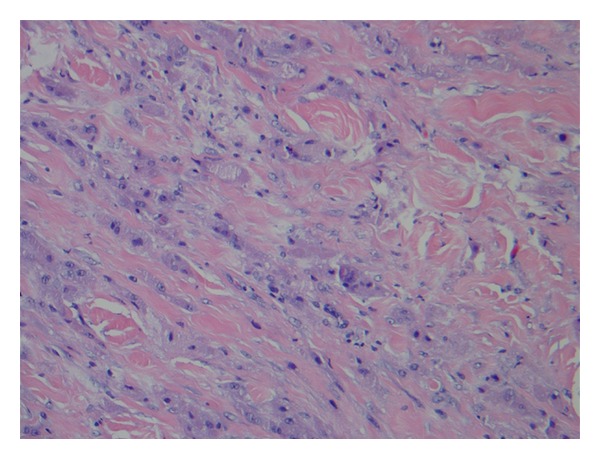
(Hematoxylin and eosin original magnification, 400x). The malignant granular cell tumor was composed of multiple large spindle cells with abundant granular cytoplasm and pleomorphic, hyperchromatic nuclei with prominent nucleoli and scattered mitotic figures.

Histological examination revealed a diffuse, poorly circumscribed neoplasm involving the available dermis and extending into the subcutaneous fat. The neoplasm was composed of multiple fascicles of large, spindle cells with abundant granular cytoplasm and pleomorphic, hyperchromatic, atypical nuclei with prominent nucleoli and scattered mitotic figures (Figure 1). Neoplastic cells were uniformly positive for S100 protein. These findings met histologic criterion for malignant granular cell tumor. The large size and relatively rapid growth of the neoplasm supported this diagnosis. The patient underwent Mohs micrographic surgery, and the tumor was excised in two stages with primary closure of the wound defect. No head and neck lymphadenopathy was found. The incision was completely healed without signs of recurrence 3 months after excision. The patient declined further work-up for malignancy.

## 2. Discussion

Granular cell tumors usually present as asymptomatic, slowly growing, poorly circumscribed nodules in adults in the fourth to sixth decade of life. They can be solitary or multiple, with a slight predominance in females and a substantial predominance in African Americans. Common sites include the head and neck, especially the tongue, although any dermal or subcutaneous site can be involved.

They have also been found in a variety of other locations, including the gastrointestinal tract [1], larynx [2], and bladder [3].

The cellular origin of granular cell tumors is controversial. Originally described as a neoplasm of muscular origin, derivation from Schwann cells or other perineural cells is considered to be most likely [4]. Localization adjacent to and within peripheral nerves supports this opinion [5]. Due to uniform staining characteristics, they are considered to be a true neoplastic entity and not the conglomeration of multiple neoplasms with focal granular cell change [4].

**Figure 2:**
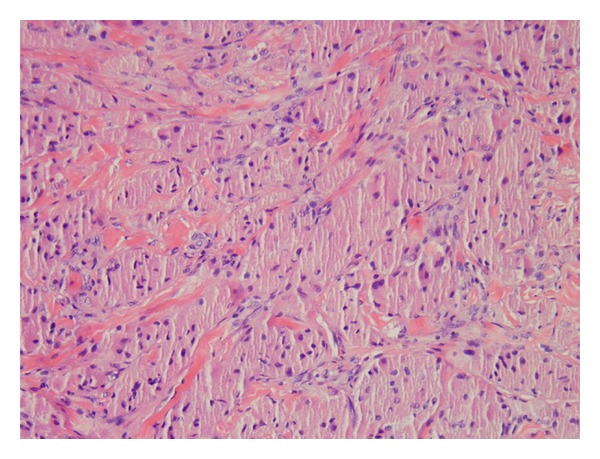
(Hematoxylin and eosin original magnification, 400x). The classic granular cell tumor, composed of a collection of polygonal or round cells with centrally placed nuclei and coarsely granular eosinophilic cytoplasm. Note the lack of nuclear atypia and mitotic figures.

Histology reveals a collection of polygonal or round cells with centrally placed nuclei and coarse eosinophilic cytoplasmic granules [6] (Figure 2). Ultrastructurally these granules have been shown to be autophagic vacuoles containing mitochondria, myelin bundles, and rough endoplasmic reticulum as well as other cellular debris [7]. The cells are arranged in nests, often divided by connective tissue septae. The cell collections can also be scattered and poorly circumscribed. There may be overlying epidermal acanthosis or pseudoepitheliomatous hyperplasia. As mentioned, granular cell tumors are often closely associated with peripheral nerves.

The malignant granular cell tumor is rare, with roughly 50 cases being reported in the literature. When compared to their benign counterparts, malignant granular cell tumors tend to have longer clinical duration with sudden rapid growth, are larger on presentation, and often involves a history of local recurrence. Reported data also includes a female predominance (70%), with presentation usually in the fifth decade of life [8, 9]. Racial predilection is difficult to determine given lack of sufficient demographic information in many reported cases. Invasion into adjacent muscle or bone on imaging or surgical pathology has been reported and is a sign of likely malignancy [10].

While many cases of malignant granular cell tumor are diagnosed after lymph node or distant metastasis, there are criteria that can differentiate this tumor from its benign counterpart with excellent clinical correlation. Malignant granular cell tumors are usually more cellular with greater variability in size and shape of cells. Recent morphologic criteria for malignancy set forth by Fanburg-Smith and colleagues include spindling of tumor cells, increased nuclear to cytoplasmic ratio, pleomorphism, necrosis, vesicular nuclei with large nucleoli, and increased mitotic activity (>2 mitoses per 10 high-powered fields at 200x magnification). The presence of 3 or more of these features strongly suggests histologic malignancy [9]. Our case displayed increased mitotic activity, spindling of tumor cells, increased nuclear to cytoplasmic ratio, and atypical vesicular nuclei with large nucleoli, meeting histologic criteria for malignancy. Mitoses were scattered and atypical but did not meet the criterion listed above. Ultrastructural features are not helpful in distinguishing between benign and malignant lesions [6].

Treatment of localized granular cell tumors, both benign and malignant, begins with surgical excision. In cases of benign granular cell tumor in which wide local excision takes place, there is 2–8% recurrence with negative margins and >20% recurrence with positive margins, emphasizing the importance of margin control [11]. In the previously mentioned cases, re-excision was not pursued when positive margins were present. Ordonez reviewed 41 cases of malignant granular cell tumor in the literature, most of which were treated with wide local excision, and found a 59% recurrence rate [12]. The frequent presentation of these tumors on the head and neck makes the conservation of tissue important. Mohs micrographic surgery, a surgical procedure which removes tumor in stages until microscopic margins are clear, is a logical choice to accomplish complete excision, conserve tissue, and minimize recurrence since the granular cell is easily recognizable with hematoxylin and eosin staining. The presence of perineural invasion can also be recognized and managed with this procedure. Mohs micrographic surgery of granular cell tumor has been described in five prior case reports, and Chilukuri et al. described excision of a granular cell tumor by Mohs micrographic surgery which was thought to be malignant due to large size and rapid growth [13–17]. Rates of recurrence of granular cell tumor after Mohs micrographic surgery are not available. Prognosis is poor in patients with malignant granular cell tumor, with frequent metastasis (>50% overall) and 30–50% mortality over three years in two case series [8, 9]. Metastatic disease is often present upon diagnosis [18]. Tumors typically spread via lymphatic and hematogenous routes to the lungs, liver, and bones. Metastasis can also become apparent long after surgical excision [19]. Treatment has been largely unsatisfactory, especially for metastatic disease. Radiation therapy and multi-agent chemotherapy generally do not improve prognosis [12].

Once diagnosed with malignant granular cell tumor, patients should undergo a full physical examination geared toward localization of metastatic disease. To exclude metastases to more commonly involved sites, some advocate screening of patients with malignant granular cell tumor with lymphatic and hepatic sonography, chest, abdominal, and pelvic computed tomography, thoracic X-ray, and bone scintigraphy [20]. The utilization of all of the aforementioned imaging modalities may yield some redundancy of imaging of sites; thus, evaluation for metastasis should be individualized based on consultation and evaluation by an oncologist. As mentioned previously, patients should have lifelong follow-up since metastases have been reported years after initial diagnosis.
